# Paclitaxel-Loaded Silk Fibroin Nanoparticles: Method Validation by UHPLC-MS/MS to Assess an Exogenous Approach to Load Cytotoxic Drugs

**DOI:** 10.3390/pharmaceutics11060285

**Published:** 2019-06-17

**Authors:** Sara Perteghella, Cristina Sottani, Valentina Coccè, Sara Negri, Loredana Cavicchini, Giulio Alessandri, Danilo Cottica, Maria Luisa Torre, Elena Grignani, Augusto Pessina

**Affiliations:** 1 Department of Drug Sciences, University of Pavia, 27100 Pavia, Italy; sara.perteghella@unipv.it; 2 Pharmaexceed S.r.l., 27100 Pavia, Italy; 3 Environmental Research Center, ICS MAUGERI SPA SB, Institute of Pavia, IRCCS, 27100 Pavia, Italy; cristina.sottani@icsmaugeri.it (C.S.); sara.negri@icsmaugeri.it (S.N.); danilo.cottica@icsmaugeri.it (D.C.); elena.grignani@icsmaugeri.it (E.G.); 4 Department of Biomedical, Surgical and Dental Sciences, University of Milan, 20100 Milan, Italy; valentina.cocce@guest.unimi.it (V.C.); loredana.cavicchini@unimi.it (L.C.); augusto.pessina@unimi.it (A.P.); 5 Department of Cerebrovascular Diseases, Fondazione IRCCS Istituto Neurologico Carlo Besta, 20133 Milan, Italy; Giulio.Alessandri@istituto-besta.it

**Keywords:** silk fibroin, nanoparticles, paclitaxel, UHPLC-MS/MS, method validation

## Abstract

The aim of this work was to load an anticancer drug, paclitaxel (PTX), on Silk Fibroin Nanoparticles (SFNs) by using an exogenous approach. SFNs were produced, freeze-dried and then loaded with PTX. An exogenous method allowed us to reduce both drug loss and environmental impact. In order to quantify PTX loaded in SFNs, a simple and reliable method using reversed phase liquid chromatography coupled to tandem mass spectrometry (rp-UHPLC-MS/MS) was developed. This methodology was validated by the determination of spiked QC samples in three consecutive days. Good accuracy and precision of the method were obtained, while the intra-day and inter-day precisions were less than 10.3%. For PTX, the limit of quantitation (LOQ) was 5.0 ng/mL. Recovery from the matrix (SFNs-PTX pellets) was calculated (81.2% at LOQ value) as PTX was entrapped in a new matrix like the polymer silk fibroin-based. This method was successfully applied to determine the encapsulation efficiency (1.00 ± 0.19%) and the nanoparticle loading (0.12 ± 0.02% *w*/*w*). The in vitro anticancer activity of SFNs-PTX was tested against CFPAC-1 cancer cells; results demonstrated a very high cytotoxic activity of SFNs-PTX, with a dose dependent inhibition of CFPAC-1 proliferation, confirmed by the IC50 value of 3450 ± 750 ng/mL.

## 1. Introduction

Nowadays nanotechnology represents one of the most promising approaches to improve the solubility and the bioavailability of poor soluble drugs [1]. In particular, many researchers have studied and developed different nanomaterials, including micelles [2,3,4], liposomes [5], nanoparticles [6,7] and nanocrystals [8]; their work aimed to improve solubility and to allow drug targeting, reducing the side effects related to the non-specific distribution of the drugs. Recently, Extracellular Vesicles (EVs) were investigated and proposed as a new category of drug delivery nanosystems [9,10]; EVs were physiologically produced by cells and could be considered as natural liposomes, composed of a phospholipid bilayer, with heterogeneous cargo such as proteins, lipids, growth factors, cytokines and miRNA. In particular, Mesenchymal Stem Cell-secreted EVs (MSC-EVs) have been of huge interest because they retain the peculiar parental cellular properties [9], such as homing and immunomodulatory ability [11]. EVs could be loaded with both “naked” [12] and nanoencapsulated drugs [13,14] using an endogenous method, before EVs isolation, or an exogenous approach, after EVs isolation [15]. These two approaches are substantially different: (i) in the endogenous method the parental cells are incubated with the selected drug and then the EVs secretion was induced; (ii) in the exogenous method, EVs are directly incubated with the drug, reducing the loss of bioactive compound and improving the encapsulation yield [15].

The same approaches could be applied to the traditional nanosystems; actually, most of published papers proposed the addiction of the selected drug during the nanoparticle’ preparation, unfortunately this approach causes the leak of drugs. In case of highly cytotoxic anti-cancer drugs, the active pharmaceutical ingredient (API) must be manipulated in adequate facilities, making the nanosystem preparation complicated. Furthermore, a loss of cytotoxic compounds must be reduced to prevent the dispersion in the environment and the possible consequent operator exposure. Based on this evidence, the exogenous loading of anti-cancer drug on nanoparticles could be advantageous; in fact, the high surface area of nanoparticles could increase the potential loading surface. Moreover, related to the cytotoxic drug manipulation problem, the exogenous approach could provide the preparation of the naked nanoparticles and then to load the API in a controlled environment (with lower drug loss and lower environmental impacts).

For these reasons, we proposed an exogenous approach to load an anti-cancer drug, paclitaxel (PTX), on silk fibroin-based nanoparticles (SFNs).

Silk fibroin (SF) is a natural protein, derived from *Bombyx mori* cocoons, which has been used for many years in biomedical fields thanks to its biocompatible and biodegradable properties [16,17,18,19]. Silk fibroin was studied as drug carriers; in particular, fibroin-based microparticles were proposed for wound healing, intra-articular delivery, and parathyroid hormone delivery applications [20,21,22,23]. Silk fibroin was also proposed for the production of nanoparticulate systems able to carry small molecules, protein, gene and anti-cancer drugs [7,24,25]. Silk fibroin can easily self-assembled in nanoparticles after exposure to salts, organic solvent, pH change and heat, and it can also be derivatized to obtain a specific targeting to the injured site [26]. Overall, many researchers have demonstrated that silk fibroin represents the ideal candidate as a drug carrier and, in addition, can be considered itself as a bioactive compound with numerous biological properties [27,28,29]. Based on these premises, we selected silk fibroin for our research.

Over the past three decades, several analytical methods based on Ultra High Performance Liquid Chromatography coupled to Mass Spectrometry (UHPLC-MS/MS) systems were developed for PTX [30,31,32,33,34,35]. All cited research was related to the quantification of PTX in biological samples (cells, plasma, urine, feces and tissues) considering the concentration range suitable for pharmacokinetic studies. Nowadays, nano-system engineering for paclitaxel bioavailability optimization (e.g., PTX bound in albumin, incorporated in poly-lactic co-glycolic acid polymers and/or entrapped in core-crosslinked polymeric micelles) is needed to develop specific analytical methods in relation to the new matrices [36,37]. As we focused on the loading of PTX on silk-fibroin nanoparticles, as a crucial factor to the nano-based system yield, a reliable methodology that involves using UHPLC-MS/MS was optimized and then validated. We envisaged that the analytical procedure would have been a more accurate approach if the method had been validated in order to assess the encapsulation efficiency and drug loading on SFNs. For example, PLGA-based nanoparticles have been reported to often present high encapsulation efficiencies with a low drug loading (around 1%) [38]. For this reason, key features and possible pitfalls in relation to the percentage of PTX loaded on these types of nanoparticles will also be discussed in this paper.

## 2. Materials and Methods

### 2.1. Materials

Reference standard Paclitaxel, PTX, (Taxol^®^), tax-11-en-9-one,5β, 20-epoxy-1,2α, 4,7β, 10β, 13α-hexahydroxy-4,10-diacetate-2-benzoate-13-(α-phenylhippurate) and the internal standard, trofosfamide (TR, 99.7% purity), were supplied by Nova Chimica (Milan, Italy) as 40 and 250 mg powder, respectively. Acetonitrile, methanol, and formic acid (HCOOH), all of LC gradient grade were purchased from VWR International Ltd. (Merck House, Poole, UK). Hydrochloric acid 37% (density, 20 °C 1.19 g/mL) and phosphate buffered saline (PBS) were obtained from VWR International Ltd. (Milan, Italy). Deionized water was generated from Milli-Q Plus water-purifying system purchased from Millipore (Milford, MA, USA). Sodium carbonate, lithium bromide and acetone were purchased from Sigma-Aldrich (Milan, Italy). Dialysis tubes were obtained from Visking (London, UK). Eppendorf tubes (15.0 mL), as well as pipette models (from P200 to P5000) were purchased from Eppendorf (Netheler-Hinz-GmbH, Hamburg, Germany). Disposable pipette tips were obtained from Rainin Instruments, Woburn, MA, USA. The centrifuge (Eppendorf^®^ Microcentrifuge 5415; Merck, NJ, USA) was used throughout the study to separate impurities from nanoformulation.

### 2.2. Silk Fibroin Extraction and Nanoparticles Preparation

To obtain the silk fibroin water solution, we started cutting the *Bombyx mori* cocoons; obtained material was boiled for 30 min in Na_2_CO_3_ solution (0.02 M) and then rinsed four times in water. After the drying of the fiber at room temperature, we dissolved the fibroin in 9.3 M LiBr solution (60 °C, 4 h) [13]. Fibroin solution was dialyzed for 72 h against water with cellulose tubes (Cut-off 3000–5000 Da). The final fibroin water solution (8% *w*/*v*) was preserved at +4 °C until the use.

SFNs were prepared as previously reported [13]. Briefly, silk fibroin aqueous solution (1.5% *w*/*v*) was added dropwise into an acetone bath, maintained under magnetic stirring, considering a volume ratio silk: acetone of 1:5. After 1 min of magnetic stirring, nanoparticle suspension was dialyzed against distilled water, using cellulose tubes (Cut-off 3–5 kDa), removing all acetone. Obtained SFNs were lyophilized (8 × 10^−1^ mbar, −50 °C for 72 h). Three SFNs batches were prepared to determine the mean production process yield (Y% = [total SFNs weight/fibroin weigh] × 100).

### 2.3. SFNs Characterization

SFNs were characterized in terms of particle size distribution, morphology by scanning electron microscopy (SEM), and physico chemical properties by Fourier Transfrm Infrared (FT-IR).

#### 2.3.1. Particle Size Distribution

Nanoparticle Tracking Analysis (NTA) was used to evaluate the SFNs size distribution (NanoSight NS500 equipment, Malvern Instruments, Malvern, UK). Each measurement was repeated 6 times (60 s each) [39]. Dynamic Light Scattering (DLS Zetasizer Nano S particle analyzer, Malvern Instruments) was used to determine the polydispersity index (PDI) of SFNs. SFNs were analyzed by ten measurements of 300 s each.

For both NTA and DLS analyses, SFNs were suspended in aqueous solution (0.5 mg/mL), sonicated and filtered (0.45 µm) before the analyses in order to eliminate nanoparticle aggregates. Three batches were analyzed to determine the reproducibility of the production method.

#### 2.3.2. Scanning Electron Microscopy (SEM)

Nanoparticle morphology was evaluated by SEM analysis (MIRA3, Tescan, Brno, Czech Republic). Freeze-dried SFNs were gold-sputter coated under argon and then analyzed. SEM analyses were performed at Arvedi Laboratory, CISRiC (Centro Interdipartimentale di Studi e Ricerche per la Conservazione del Patrimonio Culturale), University of Pavia (Pavia, Italy).

#### 2.3.3. Fourier Transform Infrared (FT-IR) Spectroscopy

FT-IR spectroscopy was selected to evaluate the secondary structure of silk fibroin in nanoparticles. SFNs spectra were obtained with Spectrum One Perkin-Elmer spectrophotometer (Perkin Elmer, Wellesley, MA, USA) coupled with a MIRacle™ ATR device (Pike Technologies, Madison, WI, USA). We considered the spectral region between 650 and 4000 cm^−1^ (resolution 4 cm^−1^); the IR spectra were recorded in transmittance mode. Each experiment was performed in triplicate.

### 2.4. Nanoparticles Loading with Paclitaxel

5 mg of SFNs were rehydrated with 1 mL of PBS for 2 h at 37 °C. After this time, SFNs were co-incubated with PTX (100 µL, 0.6 mg/mL stock solution, Kabi Fresenius Clinical Grade, Bad Homburg, Germany), at 37 °C for 2 h under magnetic stirring. SFNs-PTX were diluted in 50 mL PBS and collected by centrifugation (2.500× *g*, 10 min). This washing procedure was repeated twice to eliminate the unbounded PTX and to obtain the final pellet of SFNs-PTX. SFNs subjected to the same procedure, in the absence of PTX, were considered as controls.

### 2.5. Paclitaxel Analytical Determination

For analytical characterization, SFNs was suspended in PBS (5 mg/mL). Hydrochloric acid (HCl 37%) was diluted to the working standard conditions. The matrix solution composed of PBS-suspended SFNs, 0.12 M hydrochloric acid and mobile phase (0.1% formic acid in acetonitrile) was used to prepare daily standard calibration curves and quality control samples (QCs). The samples were centrifuged and then filtered through syringe filter devices (0.22 μm pore size, Whatman Inc., Clifton, NJ, USA).

#### 2.5.1. Chromatography

A UHPLC system is composed of an Agilent Technologies 1200 series system operating with a degasser, binary pump, and high-performance autosampler (HiP ALS SL+) with a thermostatic column compartment (Agilent 6460 Triple Quadrupole LC/MS, Santa Clara, CA, USA). The chromatographic column was a Zorbax Eclipse plus C8 2.1 × 50 mm, 1.8 m (Agilent Technologies, Inc., Santa Clara, CA, USA). The mobile phase consisted of 0.1% formic acid in water (*v*/*v*) (solvent A) and acetonitrile (solvent B). The starting mobile phase conditions were 95% A and 5% B. Then we applied a linear gradient of mobile phase B that was held for 3 min and then was increased to 60% in 4.0 min. This gradient was held for 2.0 min, followed by a reconstitution of starting conditions. The equilibration process was obtained by using 5% of B for 3.0 min, resulting in a total analysis time of 11 min. UHPLC flow rate was set at 0.4 mL/min and the column temperature to 40°C. The retention times of paclitaxel and IS were 4.5 and 3.5 min, respectively. The concentrations of paclitaxel was expressed as ng/mL of PTX, for the validation procedure, and ng of PTX, for the real samples of SFNs-PTX.

#### 2.5.2. Mass Spectrometry

The analysis was performed with the UHPLC system coupled with a 6460 quadrupole mass spectrometer (Agilent Technologies, Inc.). Equipment control, data acquisition and analysis were carried out by using an Agilent Mass Hunter workstation. 6460 Agilent and electrospray ionization (ESI) interface was used for MS/MS analysis. To enhance the sensitivity, we selected the Jet Stream technology with a super-heated sheath gas. Ions source parameters for the positive mode were as follows: vaporizer temperature, 300 °C; sheath gas, 11 mL/min with a temperature of 300 °C; nozzle voltage, 500 V and capillary voltage, 3500 V. Nitrogen was selected as nebulizer gas (35 psi, flow rate of 5 L/min). The PTX working standard solution, at the concentration of 1 µg/mL, was used to obtain the selected reaction monitoring (SRM) transitions of paclitaxel. PTX was quantified using the following transitions: *m*/*z* 854→509; 286 for PTX; *m*/*z* 323→154, for IS. SRM settings and optimization potentials were as follows: frag voltages, 75 and 123 for PTX and IS, dwell 200; collision energy 10 (arbitrary unit); accelerator voltage 7. We used a retention time window of between 2.0 and 6.0 min to adjust the scheduled SRM measurement segment.

#### 2.5.3. Stock, Working Standard Solutions and Quality Control Samples

The calibration solutions were prepared starting from the PTX standard stock solutions. The molecule was diluted in methanol (10 µg/mL). Calibration curve and quality control solutions were prepared by a serial dilution of PTX. For the calibration curve, we considered the following working standard solution concentrations: 0.10, 0.25, 0.50, 1.50, 2.50 and 5.00 µg/mL. Quality control solutions (QCs) were 0.20, 1.00 and 2.00 µg/mL. Internal standard was solubilized in methanol (0.2 µg/mL) to obtain the working solution. Solutions were stored at +4 °C in the darkness.

For PTX recovery, the stock standard solutions were prepared in mobile phase at the same concentration levels of those prepared in matrix. A mixture, composed of 100 µL of each point of these working standard solutions and 100 µL of internal standard, was vortexed and directly injected in the UHPLC-MS/MS system. During the validation study, we freshly prepared every day seven-point matrix calibration curves. Each calibration standard was obtained adding 100 µL of each working stock solution in methanol to 1 mL of matrix solution to obtain the final calibration curve for PTX with the following concentrations: 5.0, 12.5, 25.0, 75.0, 12.5, 125.0 and 250 ng/mL.

#### 2.5.4. Validation Study

The validation study was performed according to “Guidance for industry: bioanalytical method validation, FDA, US Department of Health and Human Services, 2001” [40].

#### Calibration Standards and Lower Limit of Quantification

The linearity of calibration curves was validated for different working days (*N* = 3). The least-squares linear regression equation *y = a + bx* (*y*: peak area ratio; *x*: concentration of calibration samples; *a*: intercept; *b*: slope of linear regression, obtained weighting the reciprocal of the concentration using the peak area ratio drug/internal standard) was used for the plotting of calibration curves. In order to minimize the deviation between back-calculated values and theoretical concentrations, we chosen the weighting factor. Then, QCs were prepared in a similar way at concentrations within the range of the calibration standards and were analyzed in quadruplicate (*n* = 4) on each day of the validation study. Three QCs were, therefore, at the concentrations of 10.0, 50.0 and 100.0 ng/mL. The lowest level of quantification (LLOQ) was determined by analyzing spiked samples (*n* = 4) at the concentration of 5.0 ng/mL. Moreover, each calibration curve included a blank sample (matrix processed without IS) and a zero blank sample (matrx processed with the IS).

#### Precision, Accuracy and Recovery

In order to evaluate the method precision and accuracy, we performed an intra- and inter-day validation, during three non-consecutive days. In particular, determination of intra-day accuracy and precision was performed processing QCs and comparing their calculated concentration values vs. daily calibration curves. On the other side, evaluation of inter-day accuracy and precision was carried out by analyzing each concentration of QCs in twelve replicates.

Accuracy, expressed as percentage, was assessed by the ratio between back-calculated concentration and actual value.

We considered the coefficient of variation (CV%) as measure of precision. The intra- and inter-assay accuracies should respect the recommendation, reported in the international Guidelines (2001) [40]. At each concentration, only one QC level could be excluded. The lower limit of detection (LOD) level was considered as three times the standard deviation of LC–MS/MS peak areas detected at PTX retention times. The percentage extraction recovery (RE) was calculated at the lower level of quantification for PTX and at the three concentrations of QCs. The mean integration ratio of analytes spiked in blank samples composed of nanoparticle-based matrix were compared with those obtained in the mobile phase.

#### Stability

The stability of PTX in matrix was assessed by analyzing QC samples during storage and handling. QC samples were frozen overnight at the normal storage temperature (−20 °C) and thawed unassisted at room temperature. Long-term stability was not assessed because the complex SFNs-PTX were revealed to be unstable after the first cycle of the freeze-thaw procedure.

### 2.6. Drug Loading and Loading Efficiency Evaluation

The drug loading (% *w*/*w*) of SFNs-PTX was calculated from the ratio between the total drug content (obtained from analytical determination, Section 2.5) and the concentration of analyzed nanoparticles.

Loading efficiency (EE%) was determined as percentage ratio between the actual loaded drug and the drug dissolved during SFNs-PTX preparation (Section 2.4).

### 2.7. In Vitro Potency Test of PTX, SFNs-PTX and SFNs on Cancer Cells

The anticancer activity of SFNs-PTX was in vitro tested on a human pancreatic carcinoma cell line (CFPAC-1) according to a previously described procedure [41,42]. The SFNs-PTX anti-tumoral activity was compared to that of pure drug (PTX) (CTR+) and of naked SFNs (CTR-). We tested eight concentrations for each sample; briefly, we prepared stock solutions (50 ng/mL for PTX, 750 µg/mL for SFNs and 24 µg/mL for SFNs-PTX) which were 1:2 serially diluted. CFPAC-1 cells were seeded into 96-well plate (1000 tumor cells/well) and treated with 100 µL of sample for 7 days; cell proliferation was evaluated with an MTT assay (3-(4,5-dimethyl-2-thiazolyl)-2,5-diphenyl-2-H-tetrazolium) as previously described [41]. The inhibitory concentration IC_50_ was determined according to the Reed and Muench formula [43] as the concentration of the compounds (ng/mL) able to produce a 50% inhibition of CFPAC-1 proliferation.

## 3. Results

### 3.1. SFNs Characterization

Desolvation method allowed us to minimize the loss of fibroin during the production process, as demonstrated by a high process yield of 88.56 ± 6.32% (mean ± standard deviation, *n* = 3).

NTA analyses showed a SFNs mean diameter of 115.23 ± 5.11 nm (*n* = 3), with narrow size distribution (PDI = 0.14), as confirmed by the Zetasizer analyzer. The effect of lyophilization on the SFNs size distribution was evaluated comparing the mean diameter before and after the freeze-drying procedure (111.69 ± 9.52 and 115.23 ± 5.11 nm, respectively). The lyophilization causes the nanoparticle aggregation and, to overcome this inconvenience, we sonicated and filtered the nanoparticle suspension before the use.

**Figure 1:**
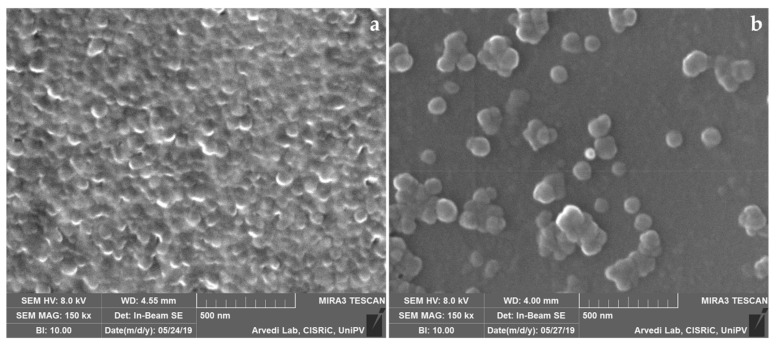
Scanning Electron Microscopy image of freeze-dried SFNs before (**a**) and after (**b**) dispersion.

SEM morphological investigation confirmed the NTA results and demonstrated that freeze-dried SFNs presented a spherical shape and smooth surface (Figure 1). Freeze-drying induced the particle aggregation (Figure 1a) but, after suspension and sonication (Figure 1b) nanoparticles appeared to be well dispersed.

FT-IR analyses were performed on lyophilized SFNs to evaluate the secondary structure of silk fibroin. Typical bands of crystalline β-sheet domains were observed in the spectra of SFNs, demonstrated that silk fibroin was in its stable conformation. In particular, we identified the bands related to amide I, amide II and amide III at about 1620, 1520 and 1230 cm^−1^, respectively (data not shown).

### 3.2. Paclitaxel Analytical Determination

#### 3.2.1. Linearity

The UHPLC-MS/MS method was validated for PTX loaded on silk fibroin-based nanoparticles over a concentration range of 5.0–250 ng/Ml considering six successive linear calibration curves (average correlation coefficients >0.998 in matrix). The average calibration curve equations obtained in mobile phase and in SFN matrix are reported in Table 1. SFN matrix was experimentally prepared along with 0.12 M hydrochloric acid, containing 0.1% formic acid in acetonitrile.

Provided for a good ratio between the slopes, the average recovery percentage of PTX was 76.5% under the optimized experimental conditions (SFNs matrix). As PTX recovery value has been considered satisfactory to release paclitaxel from the silk fibroin nanoparticles, PTX drug free was quantified in real samples (SFNs-PTX) by means of the calibration curve (*y* mean) that was prepared in SFNs matrix over the three days of the validation procedure.

#### 3.2.2. Precision, Accuracy and LOQ

Precision was evaluated at the QCs and LOQ levels within a batch (intraday) and over the three days of the validation study. The measured concentrations had precisions included between 3.21 and 8.84. Accuracy was also less than 108.2%. A complete overview of the validation parameters is detailed in Table 2. The LOQ value was set at 5 ng/mL as intra- and inter-days CV% were between 4.5 and 5.5, respectively.

#### 3.2.3. Recovery

The recovery percentages, measured at the three QCs and LOQ levels, were obtained by comparing the integration ratio values for PTX spiked in mobile phase and the same concentrations spiked in SFNs matrix. The integration values and standard deviation associated to the obtained results at the QCs and LOQ levels are summarized in Table 3. These values are in full agreement with the mean recovery value (76.5%) that was obtained by comparing the slope of the calibration standard curves.

#### 3.2.4. Stability

SFNs per HCl 0.12 M were stable at least for 8 h at room temperature in autosampler. SFNs-PTX treated and hydrolyzed with HCl were unstable at 4 °C in dark conditions overnight. Freeze and thaw cycles indicated a loss of more than 25% of the theoretical concentration at QCs levels. These results demonstrated that this type of matrix if used, it is necessary to prepare fresh samples for each analysis.

#### 3.2.5. Chromatography Mass Spectrometry

The transitions of the mass-to-charge ratio at *m*/*z* 854.1→286.2 and 854.1→509.3 were chosen for the quantitative and qualitative assessment of PTX, respectively. With the chromatography conditions described, the retention times of PTX and IS were 4.5 and 3.4 min, respectively. Thus, a total run time of 6.0 min was allowed for each injection.

**Figure 2:**
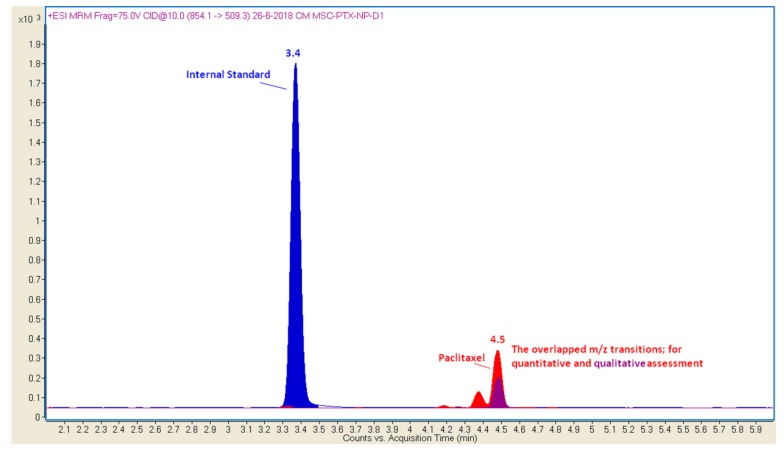
Representative SRM profiles for PTX drug free in a real sample obtained by loading the drug in silk fibroin-based nanoparticles. X-axis: retention time (minutes); Y-axis: signal intensity.

The representative SRM chromatograms for PTX drug free released by silk fibroin-based nanoparticles are reported in Figure 2.

The concentration assessed for PTX drug free in matrix solution was set at 2832.67 ± 580.001 ng/mL (mean value ± standard deviation) over the concentration range of the calibration curves obtained through the validation process. All samples were diluted before carrying out chromatographic analyses in order to obtain concentration values within the calibration curve; then, these results were properly converted by using the applied dilution factor.

### 3.3. Drug Loading and Loading Efficiency in SFNs-PTX

Results obtained from validated analytical quantification were used to determine SFNs-PTX drug loading expressed as percentage weight/weight and the loading efficiency percentage. Considering exogenous loading approach allowed us to obtain a drug loading of 0.11 ± 0.023% *w*/*w* (mean value ± standard deviation) while the loading efficiency reached 1.00 ± 0.19% (mean value ± standard deviation).

### 3.4. Potency Test on Tumor Cell Lines

The in vitro anticancer activity of free PTX, SFNs and SFNs-PTX, tested against CFPAC-1 cancer cells, was expressed as proliferation percentage referred to untreated cells considered 100%. As expected, the unloaded silk fibroin nanoparticles did not produce any pharmacological effects on CFPAC-1 proliferation and the IC50 value was estimated by extrapolation to be higher than 750,000 ± 1500 ng/mL. Free cytotoxic PTX evidenced a dramatic dose dependent inhibition of cancer cell proliferation, confirmed by the IC50 value that of 1.55 ± 1.2 ng/mL. Also, SFNs-PTX expressed a very high activity, with a dose dependent inhibition of CFPAC-1 proliferation, confirmed by the IC50 value of 3450 ± 750 ng/mL.

**Figure 3:**
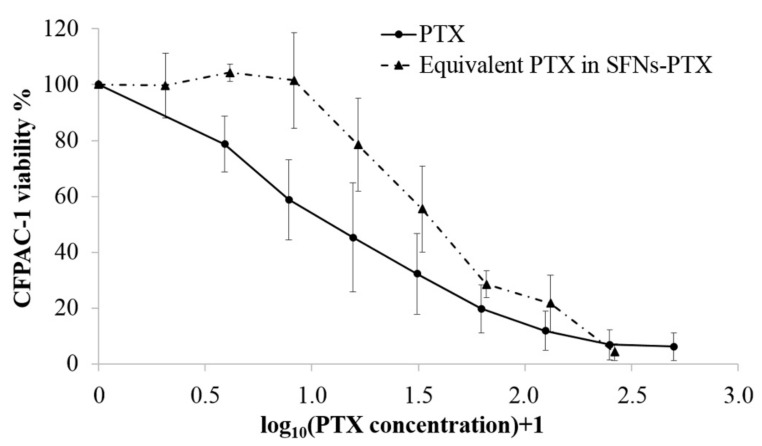
Dose/response relationship of free PTX and equivalent amount of PTX loaded in SFNs-PTX when mixed to CFPAC-1 cells. Sample concentrations (ng/mL) were expressed in the graph as log_10_(PTX concentration)+1.

To compare the PTX activity, alone or loaded in SFNs, we reported the CFPAC-1 proliferation results as function of PTX concentration. With the aim to better visually observe the differences between PTX and SFNs-PTX, the log_10_(PTX concentration)+1 was calculated (Figure 3).

Results demonstrate that PTX loaded in SFNs maintains its anti-cancer efficacy. In fact, the activity of 1.65 ng/mL of PTX loaded in SFNs (Figure 3, dotted line) does not significantly differ (*p* = 0.2394) from the same amount of free PTX (Figure 3, continuous line). The encapsulation in silk fibroin nanoparticles allowed us to maintain the pharmacological effect of paclitaxel, without any significant reduction of its in vitro effectiveness.

## 4. Discussion

We recently developed silk fibroin-based nanoparticles (SFNs) as biocompatible drug delivery systems that are able to load hydrophobic compounds [13]. Here, we proposed an exogenous method to load SFNs with paclitaxel (PTX), an anti-cancer drug characterized by high cytotoxicity. The proposed approach aimed to use lyophilized SFNs for the loading procedure, overcoming cytotoxic compound manipulation issues, such as drug loss and environmental impact.

SFNs size distribution, morphology and physico-chemical properties confirmed the data previously obtained by other researchers [13,25,44,45].

Our exogenous approach for PTX loading on SFNs allowed us to obtain a drug loading of 0.11 ± 0.023% *w*/*w* (mean value ± standard deviation). Other authors used silk fibroin nanoparticles to encapsulate PTX [25,46,47]; they proposed an endogenous method to load the drug during the nanoparticle formation process. Their approach allowed us to reach a higher drug loading, variable from 1 to 10% *w*/*w*. Wu and colleagues [25] supposed that, during the self-assembling of silk fibroin nanoparticles, PTX molecules were internalized in the hydrophobic core of nanoparticles. Differently, with our exogenous approach, PTX can react only with the silk fibroin hydrophobic blocks present on the outer surface of the nanoparticles; thus, this hypothesis could explain the low drug loading. We think that during the co-incubation of SFN and PTX, the drug is absorbed on the nanoparticle surface. Furthermore, the hydrophobic character of both PTX and silk fibroin (in its stable conformation) could induce hydrophobic interactions between these two molecules. Indeed, PTX is characterized by a partition coefficient value of Log P=3.3 and a pKa value of 10.4. It is important to take these parameters into account to let PTX molecules remain uncharged (pH = 7.0) [35]. In this condition, PTX can aggregate with silk fibroin, which is characterized by an isoelectric point (pI) ranging between 1.3 and 3.9. Therefore, the acidic pH value of the matrix was carefully studied to facilitate the precipitation of the silk protein. The experimental conditions used throughout this study based on the use of a mineral acid, such as hydrochloric acid (0.12 M) along with formic acid in acetonitrile (0.1%), were favorable to keep both the acidic environment as close as possible to the isoelectric range of this protein and to obtain PTX free compound. Hence, this optimization supports a reliable quantification of PTX with a good recovery from the hydrophobic core of these nanoparticles. In the current analytical method, under the above appropriate conditions, the UHPLC-MS/MS PTX profiles were achieved in order to obtain an accurate measurement of this highly hydrophobic molecule, loaded in a silk fibroin hydrogel. This latter is generally used in drug encapsulation processes.

According to other authors [25], we demonstrated that the encapsulation of PTX in silk fibroin nanoparticles, did not influenced its cytotoxicity profile when tested in vitro on tumor cells. This research represents a preliminary study, therefore the in vitro potency of SFNs-PTX will be tested on other cell lines to better define the effectiveness profile on a plethora of tumor models. Despite the fact that the in vitro effectiveness of PTX was not modified by the encapsulation in SFNs, we assume that our nanosystems could improve the cytotoxicity of PTX when in vivo administered. In fact, different authors demonstrated that the use of silk nanoparticles, as anticancer drug delivery systems, can increase the drug accumulation in target cancer tissues and reduce the efflux pump-mediated drug resistance [7,48]. Furthermore, silk fibroin nanoparticles can be functionalized, with the peptide motif RGD (arginine-glycine-aspartic acid), to enhance their interaction with tumor tissues and to reduce side effects [49,50]. Other experiments will be performed to optimize the exogenous drug loading procedure with the final aim to improve the drug loading and the loading efficiency. We will continue to focus our attention on fibroin nanoparticles because silk fibroin is an FDA-approved material and its use can be easily translated into clinical practice.

Further investigations will be performed to use a carrier-in-carrier drug delivery system based on the combination of SFNs-PTX and MSC-EVs. The combined use of fibroin nanocarrier and the biological vehicle (EVs) could improve the in vivo efficacy of the final product thanks to both the homing ability of the EVs and the PTX bioavailability and solubility improvement related to the fibroin nanoparticle use.

## Figures and Tables

**Table 1:** Calibration curve characteristics for quantifying PTX in SFNs-PTX.

Matrix	Conc. Range (ng/mL)	Equation	Weighting Factor
Mobile phase	5–250	*Y_mean_* = 0.00128 ± (0.002)*x* − 0.0008 ± (0.001)	1/*x*
SFN matrix	5–250	*Y_mean_*= 0.00098 ± (0.0002)*x* − 0.0022 ± (0.002)	1/*x*

**Table 2:** Intra- and inter-day validation of the method for quantitative determination of PTX.

		LLOQ	QC1	QC2	QC3
**Actual concentrations (ng/mL)**		5.00	10.00	50.00	100.00
**Intraday**					
**Measured concentrations**					
**Day1**		4.66	9.15	51.66	100.32
		4.87	9.55	48.53	102.78
		5.17	9.76	52.72	102.86
		5.07	9.29	52.59	99.55
	**Mean ± SD (QCs; *n* = 4)**	4.34 ± 0.022	9.44 ± 0.27	51.37 ± 1.95	101.37 ± 1.69
	**Precision (%)**	4.52	2.90	3.79	1.67
	**Accuracy (%)**	98.85	99.57	101.48	100.99
**Day2**		4.77	11.07	55.85	89.64
		4.67	11.82	57.57	89.78
		5.02	11.72	55.85	89.86
		4.90	11.96	54.48	99.47
	**Mean ± SD (QCs; *n* = 4)**	4.84 ± 0.16	11.64 ± 0.39	55.94 ± 0.99	92.18 ± 4.85
	**Precision (%)**	3.21	3.38	1.77	5.27
	**Accuracy (%)**	96.75	116.43	111.87	93.66
**Day3**		4.49	9.50	57.77	114.05
		4.69	9.98	52.35	100.41
		5.49	9.44	53.81	104.46
		4.99	10.43	52.63	112.98
	**Mean ± SD (QCs; *n* = 4)**	4.92 ± 0.43	9.84 ± 0.46	54.14 ± 2.80	107.98 ± 6.62
	**Precision (%)**	8.84	4.72	5.18	6.13
	**Accuracy (%)**	98.36	98.38	108.28	107.98
**Inter-day**					
**Mean ± SD (*n* = 12)**		4.90 ± 0.27	10.31 ± 1.06	53.82 ± 2.65	100.5 ± 7.60
**Precision (%)**		5.55	10.29	4.92	7.84
**Accuracy (%)**		98.00	104.80	107.60	100.50

**Table 3:** Recovery values (RE%) for PTX at QCs and LOQ levels obtained through the validation procedure.

Levels	Mean Integration Ratio ± Standard Deviation				
	Mobile Phase	CV%	Matrix	CV%	RE%
**LLOQ**	0.0046 ± 0.0005	10.3	0.0037 ± 0.0007	19.6	81.20
**QC1**	0.0093 ± 0.0011	11.4	0.0069 ± 0.0008	11.7	74.50
**QC2**	0.054 ± 0.0065	12.2	0.038 ± 0.0016	4.2	70.50
**QC3**	0.12 ± 0.11	9.8	0.099 ± 0.014	13.6	87.60

## Abbreviations

SF	Silk Fibroin
SFNs	Silk Fibroin Nanoparticles
PTX	Paclitaxel
SFNs-PTX	Silk Fibroin Nanoparticles loaded with Paclitaxel
rp-UHPLC-MS/MS	reversed phase liquid chromatography coupled to tandem mass spectrometry
LOQ	limit of quantification
LOD	limit of detection
EVs	Extracellular Vesicles
MSC-EVs	Mesenchymal Stem Cell-derived Extracellular Vesicles
API	Active Pharmaceutical Ingredient
PBS	phosphate buffered saline
SEM	Scanning Electron Microscopy
NTA	Nano Tracking Analysis
DLS	Dynamic Light Scattering
PDI	Polydispersity Index
FT-IR	Fourier Transform Infrared
QCs	Quality control samples
HiP	High-Performance Autosampler
SRM	Selected Reaction Monitoring
LLOQ	Lowest Level of Quantification
CV	Coefficient of Variation
RE	Recovery Extraction
EE	Encapsulation (Loading) Efficiency
CFPAC-1	Human pancreatic carcinoma cell line
SD	Standard Deviation
RGD	arginine-glycine-aspartic acid sequence
